# Ferrocene-Derived Pyrazinoyl and Nicotinoyl Schiff-Bases: Their Synthesis, Characterization and Biological Properties

**DOI:** 10.1155/MBD.1999.149

**Published:** 1999

**Authors:** Zahid H. Chohan, M. Praveen

**Affiliations:** 1 Department of Chemistry Islamia University Bahawalpur Pakistan; 2 Department of Chemistry Washington University St. Louis Washington 63130 USA

## Abstract

A novel class of acetylferrocene-derived Schiff-bases such as 2-pyrazinoyl-1-(2-ferroceneylmethylene)- hydrazide (HL_1_) and 2-nicotinoyl-1-(2-ferrocenylmethylene)hydrazide (HL_2_) have been synthesized and characterized by their IR, ^1^H NMR, ^13^C NMR and microanalytical date. The biological effect induced due to the coupling of ferrocene molecule with the aroylhydrazines e.g., pyrazinoylhydrazine and nicotinoylhydrazine has been studied against bacterial species such as *Escherichia coli*, *Pseudomonas aeruginosa* , *Staphylococcus aureus* and *Klebsiella pneumonae*.


### FERROCENE-DERIVED PYRAZINOYL AND NICOTINOYL SCHIFF-BASES: THEIR SYNTHESIS, CHARACTERIZATION AND BIOLOGICAL PROPERTIES

Zahid H. Chohan[*1] and M. Praveen[2]

[1] Department of Chemistry, Islamia University, Bahawalpur, Pakistan
[2] Department of Chemistry, Washington University, St. Louis 63130, USA

### ABSTRACT

A novel class of acetylferrocene-derived Schiff-bases such as 2-pyrazinoyl-1-(2-ferroceneylmethylene)-hydrazide ($HL_1$) and 2-nicotinoyl-1-(2-ferrocenylmethylene)hydrazide ($HL_2$) have been synthesized and characterized by their IR, $^1$H NMR, $^{13}$C NMR and microanalytical date. The biological effect induced due to the coupling of ferrocene molecule with the aroylhydrazines e.g., pyrazinoylhydrazine and nicotinoylhydrazine has been studied against bacterial species such as *Escherichia coli, Pseudomonas aeruginosa, Staphylococcus aureus* and *Klebsiella pneumonae*.

### INTRODUCTION

Many studies have highlighted[1-4] an extensive use of ferrocene and ferrocene-containing molecules in substituent and supramolecular chemistry, while the application of ferrocene compounds in medicinal chemistry has not been investigated at a large scale although few reports[5,6] have indicated that the replacement of aromatic group by the ferrocenyl moiety in penicillin and cephalosporines improve their antibiotic activity. Aroylhydrazines have been shown[7,8] to possess modest *in vitro* bacteriostatic properties against microorganisms such as *Mycobacterium tuberculosis*, *Mycobacterium smegmatis, Candida albicans* and *Aspergillus niger*. Preliminary studies have also shown[9,10] that such hydrazine-derived compounds are potent inhibitors of DNA synthesis in a variety of cultured human and rodent cells and their metal(II) complexes produce significant inhibition of tumor growth when given to mice bearing a transplanted fibrosarcoma[10]. Although the bioactive forms and their cytotoxic activity is equal or greater than that of many chelators previously known to possess such properties. Moreover these compounds are relatively non-toxic to mice and show some selectivity[10] in their effects. Because of these promising results we have previously synthesized many such novel aroylhydrazines or hydrazine-derived compounds and their various transition metal(II) chelates, and have tested and reported[11-15] their biological activity. Considering that interesting redox-active properties[16-18] due to $Fe^{II}$-$Fe^{III}$ already exist in ferrocene molecules, we thought it now, worthwhile to combine both the chemistry of ferrocene and already known[18-20] biologically active aroylhydrazines such as pyrazinoylhydrazine and nicotinoylhydrazineand and explore their biological properties induced by coupling with ferrocenyl group. For this purpose we have synthesized and characterized some ferrocene-derived Schiff-bases ($HL_1$ and $HL_2$) (Fig. 1) and wish to report their biological properties in this paper, which may provide a useful information and may serve as a novel potential area of research which has been ignored before.

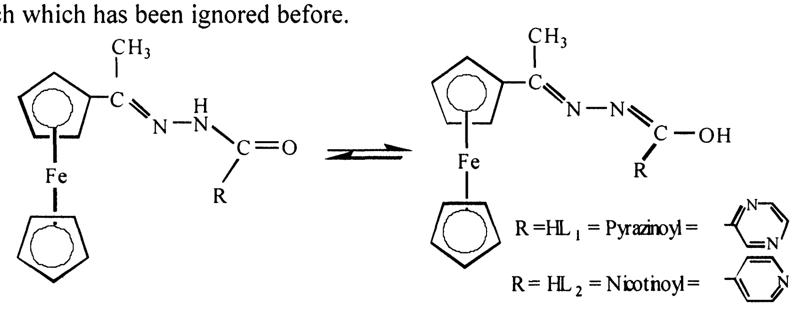

(A) (B)

Fig. 1: Structure of the Schiff-bases

### EXPERIMENTAL

### Materials and Methods

All solvents were used as Analar grade. Acetylferrocene, pyrazinoylhydrazine and nicotinoylhydrazine were obtained from Merck. IR, $^1H$ NMR and $^{13}C$ NMR spectra were recorded on Perkin Elmer 283B and 300 MHz Varian XL-300 instruments. Microanalyses were carried out by Butterworth Laboratories Ltd. Melting points were recorded on a Gallenkamp apparatus and are uncorrected.

Antibacterial studies were carried out with the help of the Microbiology Laboratory, Department of Microbiology, Qaid-e-Azam Medical College, Bahawalpur. These studies were done on wild pathogenic bacterial species collected from urine and blood samples of infected patients admitted in Bahawal Victoria Hospital, Bahawalpur.

### Synthesis of the Schiff-bases

**2-Pyrazinoyl-1-(2-ferrocenylmethylene)hydrazide ($HL_1$)**

A solution of acetylferrocene (0.01 mol) in absolute ethanol (20 mL) was added to a magnetically stirred solution of (0.01 mol) pyrazinoylhydrazine (0.01 mol) in ethanol (30 mL). The mixture was refluxed for 8 h. The dark orange solid product formed during reflux was cooled to room temperature. It was filtered, washed with ethanol and dried. The solid thus obtained was recrystallised from a mixture of hot ethanol and chloroform to give $HL_1$ (67 %), m.p 197°C. IR (KBr, $cm^{-1}$) 3215 (N-H), 1715 (C=O), 1620 (C=N), 955 (N-N). $^1H$ NMR (250 MHz, DMSO-$d^6$) δ 2.4 (s, 3H, $CH_3$), 3.8 (s, 2H, ferrocenyl), 4.1 (s, 2H, ferrocenyl), 4.3 (s, 5H, ferrocenyl), 8.5 (s, 1H, pyrazinoyl), 8.6-8.8 (dd, 2H, pyrazinoyl), 9.9 (s, 1H, NH). $^{13}C$ NMR (63 MHz, DMSO-$d^6$) δ 32.3 ($CH_3$), 68.6, 69.1, 70.6, 82.3 (ferrocenyl), 144.8,156.3, 158.8 (pyrazinoyl), 155.6 (C=N), 192.1(C=O). Analysis: Found C, 58.9; H, 4.2; N, 16.2 Calculated for $C_{17}H_{16}FeN_4O$ C, 58.6; H, 4.6; N, 16.1 %.

**2-Nicotinoyl-1-(2-ferrocenylmethylene)hydrazide ($HL_2$)**

A solution of acetylferrocene (0.01 mol) in absolute ethanol (20 mL) was added to a magnetically stirred solution of (0.01 mol) nicotinoylhydrazine (0.01 mol) in ethanol (30 mL). The mixture was refluxed for 8 h. A red solid product formed during reflux was cooled to room temperature. It was filtered, washed with ethanol and dried. The solid thus obtained was recrystallised from a mixture of hot ethanol and chloroform to yield $HL_2$ (69 %), m.p 187°C. IR (KBr, $cm^{-1}$) 3215 (N-H), 1725 (C=O), 1615 (C=N), 955 (N-N). $^1H$ NMR (250 MHz, DMSO-$d^6$) δ 2.3 (s, 3H, $CH_3$), 3.8 (s, 2H, ferrocenyl), 4.0 (s, 2H, ferrocenyl), 4.3 (s, 5H, ferrocenyl), 9.8 (s, 1H, NH), 8.7-8.9 (m, 1H, nicotinoyl), 7.8-8.0 (m, 2H, nicotinoyl), 8.3 (dd, 1H, nicotinoyl). $^{13}C$ NMR (63 MHz, DMSO-$d^6$) δ 31.9 ($CH_3$), 68.5, 69.2, 70.5, 81.9 (ferrocenyl), 155.7 (C=N), 191.8(C=O), 125.6, 138.5, 150.1, 158.8 (nicotinoyl). Analysis: Found C, 62.6; H, 4.8; N, 12.3 Calculated for $C_{18}H_{17}FeN_3O$ C, 62.3; H, 4.9; N, 12.1 %.

### Antibacterial Studies

**Preparation of Discs.**

The Schiff-base (30 μg) in DMF (0.01mL) was applied on a paper disc [prepared from blotting paper (3 mm diameter)] with the help of a micropipette. These discs were left in an incubator for 48 h at 37° C and then applied on the bacteria grown agar plates.

**Preparation of Agar Plates.**

Minimal agar was used for the growth of specific bacterial species. For the preparation of agar plates for *Escherichia coli*, MacConkey agar (50 g), obtained from Merck, was suspended in freshly distilled water (1 L). It was allowed to soak for 15 minutes and then boiled on a water bath until the agar was completely dissolved. The mixture was autoclaved for 15 minutes at 120° C and then poured into previously washed and sterilized Petri dishes and stored at 40° C for inoculation.

**Procedure of Inoculation.**

Inoculation was done with the help of a platinum wire loop which was made red hot in a flame, cooled and then used for the application of bacterial strains.

**Application of Discs.**

A sterilized forceps was used for the application of paper discs on the already inoculated agar plates. When the discs were applied, they were incubated at 37° C for 24 h. The diameter of the zone of inhibition was then measured.

### RESULTS AND DISCUSSION

The Schiff-bases $HL_1$ and $HL_2$ (Fig 1) were prepared by a simple condensation reaction. Both of them are soluble in polar solvents such as methanol, ethanol and DMF, but are insoluble in weakly polar or nonpolar solvents. The structural determination of these Schiff-bases was done with the help of their IR, $^{1}H$ NMR, $^{13}C$ NMR and microanalytical data.

The IR spectra of the $HL_1$ and $HL_2$ showed well-defined ν(C=O) and ν(N-H) modes and no stretching due to the presence of ν(OH) frequency in the region at 3365-3420 $cm^{-1}$ was found indicative of their probable keto form (Fig. 1A) than the enol form (Fig. 1B). The important IR frequencies of $HL_1$ showed some characteristic bands at 3215, 1715, 1620 and 955 $cm^{-1}$. These were assigned[19,20] to ν(N-H), ν(C=O), ν(C=N) and ν(N-N) stretches respectively. Similarly, the IR spectra of $HL_2$ showed characteristic absorption bands at 3215, 1725, 1615, 1550 and 955 $cm^{-1}$ assigned[21] to ν(N-H), ν(C=O), ν(C=N), ν(C=C) and ν(N-N) stretches respectively. The disappearance of the band at 3160 $cm^{-1}$ in both the Schiff-bases due to ν($NH_2$) and appearance of a new band at 1615-1620 $cm^{-1}$ due to Schiff-base azomethine linkage[22] confirmed the formation of $HL_1$ and $HL_2$. The $^{1}H$ NMR and $^{13}C$ NMR spectra also displayed signals assignable to all other carbons and hydrogens expected in their region respectively[23]. Also, the absence of phenolic protons in the $^{1}H$ NMR spectra confirmed the keto form configuration of these Schiff-bases. The $^{1}H$ and $^{13}C$ signals of feerocene moiety were assigned by comparing their shifts with the experimental evidences[24,25]. Furthermore, the microanalytical data of C, H and N confirmed their proposed structures (Fig. 1A).

**Antibacterial Properties**

Antibacterial properties of $HL_1$ and $HL_2$ in comparison to the simple acetylferrocene ($L_3$) were studied against bacterial species *Escherichia coli, Pseudomonas aeruginosa, Staphylococcus aureus* and *Klebsiella pneumonae*. These were tested at a concentration of 30 μg/0.01 mL in DMF solution using a paper disc diffusion method devised and reported[26,27] earlier by us. The results of these studies reproduced in Table 1 indicated that acetylferrocene and both the Schiff-bases ($HL_1$ and $HL_2$) showed variable activity against one or more bacterial strains. In comparison to acetylferrocene ($L_3$), the Schiff-base derivatives ($HL_1$ and $HL_2$) were found to be more biologically active. These studies however, provided a useful information about the biological activity of ferrocene-containing compounds and the knowledge that this activity/potency could become more pronounced when more potent compounds are coupled with ferrocene molecule and thus introduce a new potential class of biologically active compounds.

Table 5 Antibacterial Activity Data

| Ligands | Microbial Species | | | |
|---|---|---|---|---|
| | a | b | c | d |
| $HL^1$ | +++ | ++++ | +++ | +++ |
| $HL^2$ | ++++ | +++ | ++ | +++ |
| $L^3$ | ++ | + | - | + |

a=*Escherichia coli*, b= *Staphylococcus aureus*,
c= *Pseudomonas aeruginosa* d=*Klebsiella pneumonae*
Inhibition zone diameter mm (% inhibition): +, 6-10 (27-45 %); ++, 10-14 (45-64 %); +++, 14-18 (64-82 %); ++++, 18-22 (82-100 %). Percent inhibition values are relative to inhibition zone (22 mm) of the most active compound with 100 % inhibition.

ACKNOWLEDGEMENT

The authors gratefully acknowledge the Department of Pathology, Qaid-e-Azam Medical College, Bahawalpur, for its help in undertaking the antibacterial studies.

REFERENCES

1. M. L. H. Green, *Polyhedro.n*, **11**, 1489 (1992).
2. J. M. Lehn, *Angew. Chem., Int. Ed. Eng.*, **29**, 1304 (1990).
3. B. J. Coe, C. J. Jones, J.A. McCleverty, D. Bloor, P. V. Kolinsky and R. Jones, *J. Chem. Soc., Chem. Commun.*, 1485 (1989).
4. A. C. Constable, *Angew. Chem., Int. Ed. Eng.*, **30**, 407 (1991).
5. E. I. Edwards, R. Epton and G. Marr, *J. Organomet. Chem.*, **85**, C23 (1975).
6. B. W. Rockett and G. Marr, *J. Organomet. Chem.*, **123**, 205 (1976).
7. H. A. Offe, W. Siefken and G. Domagk, *Z. Naturforsch.*, **7**, 446 (1952).
8. J. R. Dimmock, G. B. Baker and W. G. Taylor, *Can. J. Pharm. Sci.*, **7**, 100 (1972).
9. D. K. Johnson, T. B. Murphy, N. J. Rose, W. H. Goodwin and L. Pickart, *Inorg. Chim. Acta.*, **67**, 159 (1982).
10. L. Pickart, W. H. Goodwin, W. Burgua, T. B. Murphy and D. K. Johnson, *Biochem. Pharmacol.*, **32**, 3868 (1983).
11. Z. H. Chohan and A. Rauf, *Synth. React. Inorg. Met-Org. Chem.*, **26**, 591 (1996).

12. Z. H. Chohan, M. Praveen and S. K. A. Sherazi, *Metal-Based Drugs.*, **5**, 267 (1998).
13. Z. H. Chohan and S. K. A. Sherazi, *Metal-Based Drugs.*, **4**, 69 (1997).
14. Z. H. Chohan and S. K. A. Sherazi, *Metal-Based Drugs.*, **4**, 65 (1997).
15. Z. H. Chohan and S. K. A. Sherazi, *J. Chem. Soc. Pak.*, **19**, 169 (1997).
16. Y. Ueno, H. Sano, M. Okawara, *J. Chem. Soc., Chem. Commun.*, 28 (1980).
17. E. Cerrada, M. R. Bryce and A. J. Moore, *J. Chem. Soc., Perkin. Trans.*, **1**, 537 (1993).
18. A. J. Moore, P. J. Skabara, M. R. Bryce, A. S. Batsanov, J. A. K. Howard and S. T. A. K. Daley, *J. Chem. Soc., Chem. Commun.*, 417 (1993).
19. J. E. Kovacic, *Spectrochim Acta.*, **23A**, 183 (1967).
20. R. A. Condrate and K. Nakamoto, *J.Chem.Phys.*, **42**, 2590 (1965).
21. J. F. Jackowitz, J. A. Durkin and J. G. Walter, *Spectrochim.Acta.*, **23A**, 67 (1967).
22. F. Jackowitz and J. G. Walter, *Spectrochim.Acta.*, **22**, 1393 (1966).
23. D.H.Williams and I.Fleming,"Spectroscopic Methods in Organic Chemistry", 4th Ed, McGraw Hill, London (1989).
24. Z. H. Chohan, Thesis, "Synthesis of Heterocycles via Ferrocene Derivatives", University of Strathclyde, U.K, 1983.
25. Z. H. Chohan, Thesis, "Co-ordination Chemistry of the 1,3-Dithiole-2-thione (DMIT) and 2-One-4,5-dithiolato (DMIO) Compounds", University of Aberdeen, U.K, 1997.
26. Z. H. Chohan and A. Rauf, *J. Inorg. Biochem.*, **46**, 41 (1992).
27. Z. H. Chohan and S. Kausar, *Chem. Pharm. Bull.*, **40**, 2555 (1992).

**Received: March 29, 1999 - Accepted: April 21, 1999 - Received in revised camera-ready format: April 27, 1999**

